# Comparing emergency department use among individuals with varying levels of cognitive impairment

**DOI:** 10.1186/s12877-022-03093-5

**Published:** 2022-05-02

**Authors:** Rebecca K. Green, Manish N. Shah, Lindsay R. Clark, Robert J. Batt, Nathaniel A. Chin, Brian W. Patterson

**Affiliations:** 1 BerbeeWalsh Department of Emergency Medicine, University of Wisconsin-Madison, 800 University Bay Drive Suite 310, Madison, WI 53705 USA; 2 Department of Medicine, Division of Geriatrics and Gerontology, University of Wisconsin-Madison, Madison, WI USA; 3 Department of Population Health Sciences, University of Wisconsin-Madison, Madison, WI USA; 4 Geriatric Research Education and Clinical Center, William S Middleton Memorial Veterans Hospital, Madison, WI USA; 5 Wisconsin School of Business, University of Wisconsin - Madison, Madison, WI USA; 6 University of Wisconsin Health Innovation Program, Madison, WI USA; 7 Department of Industrial and Systems Engineering, University of Wisconsin-Madison, Madison, WI USA; 8 Department of Biostatistics and Medical Informatics, University of Wisconsin-Madison, Madison, WI USA

**Keywords:** Dementia, ADRD, Mild cognitive impairment, Health care utilization

## Abstract

**Introduction:**

As the population ages, Alzheimer’s disease and related dementias (ADRD) are becoming increasingly common in patients presenting to the emergency department (ED). This study compares the frequency of ED use among a cohort of individuals with well-defined cognitive performance (cognitively intact, mild cognitive impairment (MCI), and ADRD).

**Methods:**

We performed a retrospective cohort study of English-speaking, community-dwelling individuals evaluated at four health system-based multidisciplinary memory clinics from 2014–2016. We obtained demographic and clinical data, including neuropsychological testing results, through chart review and linkage to electronic health record data. We characterized the frequency and quantity of ED use within one year (6 months before and after) of cognitive evaluation and compared ED use between the three groups using bivariate and multivariate approaches.

**Results:**

Of the 779 eligible patients, 89 were diagnosed as cognitively intact, 372 as MCI, and 318 as ADRD. The proportion of subjects with any annual ED use did not increase significantly with greater cognitive impairment: cognitively intact (16.9%), MCI (26.1%), and ADRD (28.9%) (*p* = 0.072). Average number of ED visits increased similarly: cognitively intact (0.27, SD 0.72), MCI (0.41, SD 0.91), and ADRD (0.55, SD 1.25) (*p* = 0.059). Multivariate logistic regression results showed that patients with MCI (odds ratio (OR) 1.62; CI = 0.87–3.00) and ADRD (OR 1.84; CI = 0.98–3.46) did not significantly differ from cognitively intact adults in any ED use. Multivariate negative binomial regression found patients with MCI (incidence rate ratio (IRR) 1.38; CI = 0.79–2.41) and ADRD (IRR 1.76, CI = 1.00–3.10) had elevated but non-significant risk of an ED visit compared to cognitively intact individuals.

**Conclusion:**

Though there was no significant difference in ED use in this small sample from one health system, our estimates are comparable to other published work. Results suggested a trend towards higher utilization among adults with MCI or ADRD compared to those who were cognitively intact. We must confirm our findings in other settings to better understand how to optimize systems of acute illness care for individuals with MCI and ADRD.

**Supplementary Information:**

The online version contains supplementary material available at 10.1186/s12877-022-03093-5.

## Introduction

Alzheimer’s disease and related dementia (ADRD) are a growing public health problem which affects an estimated 13.9% of the US population over the age of 70 [1]. This is of particular concern as ADRD is a disease of aging and its prevalence will continue to rise with the aging population [2]. Persons with ADRD suffer from a wide range of cognitive, behavioral, and psychological deficits including memory loss, confusion, irritability, and mental decline [3]. They are also at greater risk for progressive functional decline in activities of daily living including eating, bathing, transferring, and toileting [4]. Such symptoms make these individuals particularly prone to increased use of the health care system [5].

Current literature suggests some differences in ED use rates among individuals with ADRD, however these studies have either focused on health care systems outside the U.S., used purely administrative data, or relied on brief assessments without detailed neuropsychological testing to identify these patients. These studies have shown that community-dwelling persons with ADRD are 35–75% more likely to have an ED visit in a year than community-dwelling persons without ADRD [5–8]. Additionally, ED patients with ADRD are 27–37% more likely than patients without ADRD to have at least one ED visit in the 30 days following an initial ED visit [7, 8].

Unfortunately, current US-based studies all suffer from potential misclassification of subjects’ underlying cognitive status, as no study has used neuropsychological testing to definitively characterize the cognitive status of their population. Ascertaining true ADRD status requires a battery of neuropsychological tests, and this “gold” standard diagnostic is costly and time consuming. ADRD status is both underdiagnosed and underreported in the medical record, so studies that rely solely on this method lack an adequate comparison group of patients who are known to be cognitively intact, contributing to misclassification bias in the literature [9]. Additionally, published studies do not separately identify individuals with mild cognitive impairment (MCI) and screening tests are not very reliable or accurate in identifying people who have MCI. Misclassifying these individuals into either the cognitively intact or ADRD groups could significantly alter the ED use rates for those groups.

In this study, we sought to characterize ED use among a pre-existing cohort of cognitively well-characterized individuals and compare utilization rates between older adults with normal cognition, MCI, and ADRD. Through this work, we aimed to provide insight into how ED use may vary with cognitive performance. We hypothesized that worsening cognitive performance would be associated with more frequent ED use.

## Methods

### Study design

We performed a retrospective observational cohort study by reviewing electronic health records (EHRs) and linking abstracted information to a curated, research-quality database of patient and administrative data. This study was reviewed and found to be exempt by the University of Wisconsin-Madison Institutional Review Board, and a waiver of informed consent was granted.

### Study setting and population

**Fig. 1:**
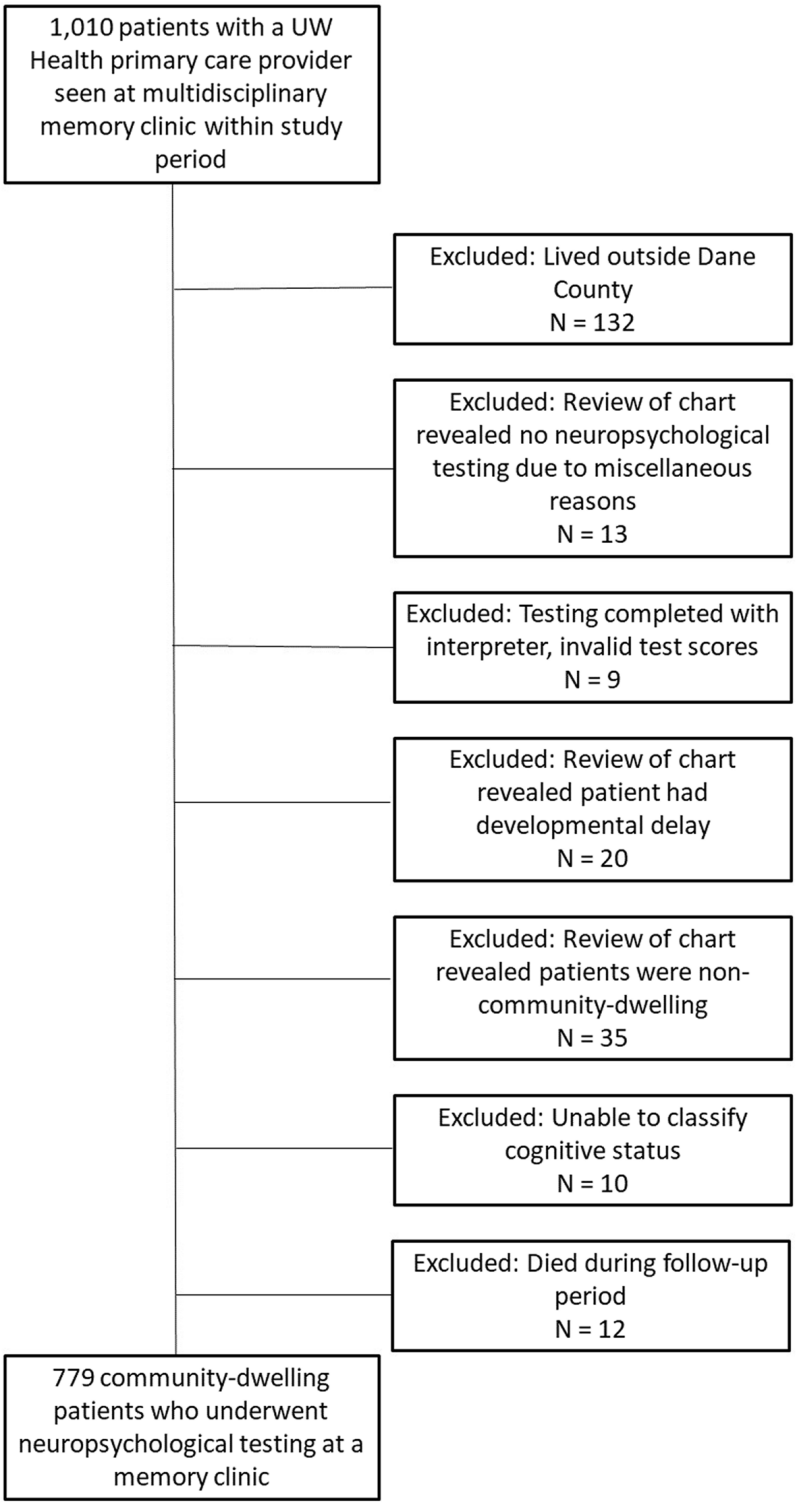
Subject Selection Diagram

We included all community-dwelling adult patients who visited one of four multidisciplinary memory clinics from 2014–2016. Any individual can be seen in these clinics, but individuals tend to be referred for evaluation of subjective memory complaints. We only included Dane County, Wisconsin residents with a University health system-affiliated primary care provider to maximize the quality of health and health utilization data. This is because previous assessments of our health system have indicated that these patients are significantly less likely to seek emergency care in either of our EDs, therefore we would not have outcome data on them. Of note, the health system participated in accountable care organization contracts (ACO) aiming to deliver coordinated, high quality care to a population while sharing financial risk. We excluded patients with developmental delay or who completed testing using a translator, due to neuropsychological testing validity issues, and patients who died during the follow-up period, as they did not contribute a full year to the analysis time. Figure 1 details the number of subjects excluded for each criterion.

The final dataset for analysis included 779 patients in which a full neuropsychological testing battery was performed. ED visits were captured from two EDs: an academic medical center with level 1 trauma center accreditation that sees approximately 60,000 patient visits per year and one community hospital that is part of the same health system. The insurance and health system structure in Madison, Wisconsin leads to residents primarily receiving ED care from their in-network hospitals.

### Study protocol

With the list of research subjects, the primary author (RKG) performed a retrospective chart review guided by best practices outlined by Kaji and colleagues [10] (Additional file 1: Appendix 1). We used HIPAA best practices for privacy and security of the data for research purposes. RKG abstracted scores from psychological testing and verbatim diagnoses by the neuropsychologist from the medical record using a data form developed by the study team through iterative revisions and pilot testing. The primary author was trained to abstract the charts by a senior author (MNS) and neuropsychologist (LRC). The first ten abstracted records were reviewed independently by an experienced physician (MNS) for accuracy, with additional batches of five abstracted records until it was determined that there were no errors and that the chart reviewer was confident in performing the abstractions accurately. Questions that came up during chart review were flagged and brought to the experienced physician (MNS) and the neuropsychologist (LRC) for clarification.

We then merged the abstracted data with ED use data from the health system’s electronic health record. We electronically abstracted health care use data and detailed subject data available in discrete fields, including the primary outcome variable, from an institutional EHR database curated for research purposes. We performed exploratory data analysis on all variables of interest to check for outliers and to ensure valid data after cleaning.

### Measurements

We abstracted the neuropsychologist and physician notes from each memory clinic visit for demographic and clinical variables including education level, living status (i.e. community dwelling or not), Mini Mental State Examination (MMSE) [11], Clock Draw [12], Semantic Fluency [13], Trails A [14], Trails B [14], Repeatable Battery for the Assessment of Neuropsychological Status (RBANS) [15], and Cognistat [16] scores. The clinical diagnostic impression was also abstracted, which was our primary independent variable. Subjects were classified as being Cognitively Intact, having Mild Cognitive Impairment, or having ADRD based on the clinical diagnostic impression from the encounter. Any subjects without clear cognitive classification were reviewed by the neuropsychologist (LRC) and excluded if no determination could be reached. Additional covariates included age, sex, race/ethnicity, education level, and number of Elixhauser comorbidities [17].

The primary outcome variable was the number of ED visits occurring within 182 days before and after a memory clinic visit. We chose the timeframe before and after the memory clinic visit to capture a time throughout which patients were likely to have the same cognitive status.

## Data analysis

All analyses were conducted using Stata, version 15.1 (Statacorp LLC, College Station, Texas). Descriptive statistics detailing population age, sex, race, education level, number of comorbidities, and MMSE score are listed in Table 1. We performed a Chi-squared test to compare the proportion of each group that used the ED and a Kruskal–Wallis test to compare the number of ED visits in each group. We used the Kruskal–Wallis test as opposed to a parametric test given unequal variances between groups. Age, sex, race, education level, and number of comorbidities were determined to be important covariates a priori because they are common influencers of healthcare [18]. We assessed additional covariates, including clinic and provider characteristics, through pairwise comparisons and found no significant differences between the groups. We performed a post-hoc power calculation using G*Power based on the F-distribution, which yielded 63% power at α = 0.05 with the given sample size and group distribution.

**Table 1:** Sample Characteristics by Cognitive Status

Characteristics	Total *N* = 779 Frequency (Relative Frequency)	Cognitively Intact *N* = 89 Frequency (Relative Frequency)	MCI *N* = 372 Frequency (Relative Frequency)	ADRD *N* = 318 Frequency (Relative Frequency)
Age				
Mean (SD)	76.3 (8.7)	72.1 (7.8)	75.9 (8.4)	78.0 (8.8)
Sex				
Male	331 (42.49)	35 (39.33)	178 (47.85)	118 (37.11)
Female	448 (57.51)	54 (60.67	194 (52.15)	200 (62.89)
Race				
White	741 (95.61)	88 (98.88)	357 (96.75)	296 (93.38)
Non-white	34 (4.39)	***	12 (3.25)	21 (6.62)
Education				
< 12 years	51 (6.55)	3 (3.37)	216 (58.06)	34 (10.69)
12 years	172 (22.08)	15 (16.85)	68 (18.28)	89 (27.99)
13–15 years	142 (18.23)	12 (13.48)	74 (19.89)	56 (17.61)
16 + years	414 (53.15)	59 (66.29)	216 (58.06)	139 (43.71)
Elixhauser Comorbidity Score				
Mean (SD)	2.8 (2.0)	2.8 (2.1)	2.7 (1.9)	2.8 (1.9)
MMSE				
Mean (SD)	25.3 (4.1)	28.9 (1.2)	26.8 (2.3)	22.5 (4.4)
ED Visits within one year of memory clinic visit				
Proportion of Population with 1 + ED Visit (95%CI)*	0.26 (0.23–0.29)	0.17 (0.10–0.26)	0.26 (0.22–0.31)	0.29 (0.24–0.34)
Median ED Visits (IQR)*	0 (0, 1)	0 (0, 0)	0 (0, 1)	0 (0, 1)

^*^p < 0.1

*ADRD* Alzheimer’s Disease and Related Dementia, *SD* Standard Deviation, *MMSE* Mini Mental State Examination, *ED* Emergency Department, *IQR* Interquartile Range

We constructed a multivariate logistic regression model to control for pre-specified covariates to generate odds ratios (ORs) for the likelihood of any ED visit occurring by cognitive performance. This model was chosen due to the binary outcome of interest and was evaluated using Hosmer-Lemshow and Pearson goodness of fit tests, which both indicated appropriate model fit. We also performed a negative binomial regression to compare the count per year of ED visits between groups. This model was chosen due to overdispersion of the data (i.e. too many zero counts to perform a Poisson regression). We chose to conduct both analyses to first see if there was a difference in the proportion of patients seeking ED care between groups and then to evaluate for differences in the quantity of ED visits between groups.

## Results

### Sample characteristics and between-group comparisons

Of the 779 patients in the final analysis, the mean age was 76.3 years (standard deviation (SD) 8.7), the majority (57.5%) were female, 95.6% were white, and 53.2% had a college degree or more, which is consistent with Dane County’s demographics. Additionally, 72.4% of the population had two or more comorbidities. The median number of ED visits within one year of memory clinic assessment was 0 (Interquartile Range (IQR) 1). See Table 1 for full characterization of the cohort, both as a whole and by cognitive status. As expected, individuals with diagnosed cognitive impairment tended to be older and have lower average Mini Mental State Exam (MMSE) scores: cognitively intact (28.9, SD 1.2), mild cognitive impairment (26.8, SD 2.3), and ADRD (22.5, SD 4.4). The number of patients having one or more ED visits during the follow-up period did not significantly increase with greater cognitive impairment: cognitively intact (16.9%), mild cognitive impairment (26.1%), and ADRD (28.9%). The Chi-squared test yielded a *p* = 0.072.

Table 2 presents population characteristics by ED use. As expected, individuals with more ED visits tended to be older and have more comorbidities, though MMSE score did not differ.

**Table 2:** Sample Characteristics by ED Utilization

Characteristics	0 ED Visits *N* = 575 Frequency (Relative Frequency)	1 ED Visit *N* = 129 Frequency (Relative Frequency)	2 + ED Visits *N* = 75 Frequency (Relative Frequency)
Cognitive Status			
Intact	74 (12.87)	10 (7.75)	5 (6.67)
MCI	275 (47.83)	65 (50.39)	32 (42.67)
ADRD	226 (39.30)	54 (41.86)	38 (50.67)
Education			
< 12 years	39 (6.78)	8 (6.20)	4 (5.33)
12 years	129 (22.43)	30 (23.26)	13 (17.33)
13–15 years	98 (17.04)	27 (20.93)	17 (22.67)
16 + years	309 (53.74)	64 (49.61)	41 (54.67)
Age			
Mean (SD)	75.9 (8.5)	77.5 (9.1)	77.9 (9.0)
Sex			
Male	242 (42.09)	59 (45.74)	30 (40.00)
Female	333 (57.91)	70 (54.26)	45 (60.00)
Race			
White	545 (95.45)	127 (98.45)	69 (92.00)
Non-white	26 (4.55)	2 (1.55)	6 (8.00)
Elixhauser Comorbidity Score			
Mean (SD)	2.6 (1.9)	3.0 (1.9)	3.8 (2.1)
MMSE			
Mean (SD)	25.4 (4.2)	25.1 (3.9)	25.2 (3.7)

*ED* Emergency Department, *MCI* Mild Cognitive Impairment, *ADRD* Alzheimer’s Disease and Related Dementia, *SD* Standard Deviation, *MMSE* Mini Mental State Examination

### Multivariate analysis

We constructed two multivariate regression models to evaluate the effect of cognition for both the presence and rate of ED use. Covariates included in both models were age, sex, race, education, and number of comorbidities. The results of both models are presented in Table 3.

**Table 3:** Odds Ratios and Incidence Rate Ratios from Multivariate Regression

Group	Odds Ratio (95% CI)	Incidence Rate Ratio (95% CI)
ADRD	1.84 (0.98 – 3.46)	1.76 (1.00 – 3.09)
MCI	1.62 (0.87 – 3.00)	1.38 (0.79 – 2.41)
Cognitively Intact	Reference	Reference

*CI* Confidence Interval, *MCI* Mild Cognitive Impairment, *ADRD* Alzheimer’s Disease and Related Dementia

Multivariate logistic regression found patients with MCI (OR 1.40; CI = 0.81–2.42) and ADRD (OR 1.50; CI = 0.86–2.61) to have greater odds (point estimate) of any ED visit compared to cognitively intact, but this difference was not statistically significant.

Multivariate negative binomial regression found patients with MCI (IRR 1.38; CI = 0.79–2.41) and ADRD (IRR 1.76; CI = 1.00–3.10) to have greater rates (point estimate) of using the ED compared to cognitively intact older adults, but this difference was not statistically significant.

## Discussion

This study compared ED use among patients based on cognitive status as defined by rigorous neuropsychological testing in an existing cohort of patients. In both analyses, we did not find increased ED use in adults with worse cognition (cognitively intact to MCI to ADRD), though we were likely underpowered to detect a statistically significant difference. Our findings in this small but well-characterized group of patients support ED utilization estimates published in the literature. In our cohort, we found that the rate of ED use for patients with MCI was 37.1% greater than cognitively intact patients, which is in line with what has been previously reported [19], though few studies have looked at this population in particular. Previous studies comparing the proportion of ED visits among patients with and without ADRD have shown that patients with ADRD use the ED anywhere from 35%-49% more often than patients without ADRD [6, 7, 19, 20] while patients with MCI use the ED approximately 35% [19] more often than cognitively intact patients. Our results are within the range reported by those studies that have looked at patients with and without ADRD, suggesting patients with ADRD use the ED 48.5% more often than patients without ADRD.

Of note, our rates of ED use may have been reduced due to the health system’s participation in an ACO. Participants in ACOs with ADRD have reduced rates of preventable ED visits than those not participating in ACOs, potentially because they have systems in place to avoid ED care [21]. Additionally, it’s possible that these patients were less likely to require ED care than patients identified purely through administrative data because of their access to memory clinic resources. It will be important to replicate this study in other communities and populations with the presence of a truly negative control group, because using solely ICD codes in the health record can have highly variable reliability [9, 22], to further elucidate these trends.

We know that ED visits, many of which are preventable, can place undue burden on patients and their caregivers as well as the health care system. A trip to the ED can be a harbinger for potential problems in patients with ADRD, as this environment can often exacerbate underlying challenges and can be incredibly disorienting for the patient and their family [23–26]. Yet, older adults have disproportionately more preventable ED visits each year than their younger counterparts [27]. For patients with cognitive impairment, however, this disparity appears to be even larger. Although studies have shown that ED use increases during the last year of life for people with ADRD [28], little is known about ED use earlier in patients’ clinical course. These results suggest that patients with cognitive impairment, especially patients with ADRD, use the ED more frequently than cognitively intact patients, which puts them at risk for poor health outcomes and further decline.

If we are able to understand what brings these patients to the ED, we will be better equipped to develop alternative ways of delivering emergency care to this unique patient population and improving the ED experience for these patients. Within the older adult population, there are several factors that prompt patients and their caregivers to seek emergency care [29]. For those with ADRD in particular, several studies have suggested that both caregivers and primary care providers play an important role in the decision to use the ED [30, 31]. Caregivers of patients with ADRD and primary care providers who treat these patients often feel ill-equipped to properly manage their acute care needs, which could be contributing factors causing these patients to seek emergency care [30–32]. The ED, however, is seldom the best place to treat patients with ADRD. If we can better understand the needs of this patient and caregiver population, we can craft treatment mechanisms that address these factors, such as community paramedicine or tailored support from primary care, to help patients avoid the ED and potential poor outcomes associated with it.

When ED visits are unavoidable, care must be taken to tailor emergency care for ADRD patients. It is difficult for patients with cognitive impairment to cope with the noise and pace of care in the ED, which can overwhelm the patient and caregiver, leading to poor health outcomes and dissatisfaction with care [26]. Additionally, we know that general older ED patient adherence to discharge instructions, such as medication changes, is poor, [33] and additional barriers of cognitive impairment likely compound this problem, increasing the likelihood of return visits to the ED [34]. Staff expertise in caring for these patients and environmental factors can directly affect the ED experience for these patients, and modifications in these areas may improve these encounters [26]. It is critical to address these contextual factors to improve ED visits and outcomes for cognitively impaired patients.

Addressing the needs of this population will require additional work. First, examining how the ED workup and outcomes differ between patients with ADRD, MCI, and older adults who are cognitively intact could help identify risk factors or patterns for poor outcomes after an ED visit. Examining the rate of recidivism and care trajectories between groups after they leave the ED will help identify vulnerable points in the patient care timeline at which additional support may be beneficial. Finally, it is crucial to understand caregiver and physician perspectives regarding treatment and management of these patients so that interventions are tailored to the needs of the population.

### Limitations

Our single center design may make the results less generalizable to the population as a whole, as our cohort of patients who received memory clinic evaluations is comprised predominantly of white, highly educated patients. Furthermore, we only captured ED use if patients visited an ED in our health system, thus if a patient sought care outside of our health system, we did not have a way of capturing that utilization. This suggests we potentially underestimated overall ED use; however the risk of this introducing bias into our analysis is low as we have no reason to suspect that cognitively intact patients seek care outside the health system at a different rate than patients with ADRD. We were also limited by the disproportionately low number of cognitively intact controls identified and included in this analysis, which greatly reduced the power of this study. Finally, given our retrospective chart review study design, we were unable to capture some potentially informative covariates; for example functional status, a critical issue for older adults and among individuals with ADRD, was not documented in the health record and depression testing results were not consistently documented.

## Conclusion

We did not find a statistically significant difference in ED use among older adults with cognitive impairment, though our point estimates are consistent with other findings in the literature. This study should be replicated in a larger cohort of cognitively well-characterized patients to confirm or refute the trends we found.

## Supplementary Information

**Additional file 1:** **Appendix 1.** Methods for Looking Through the Retrospectoscope.

## Data Availability

The datasets generated and/or analyzed during the current study are not publicly available due to privacy and ethical concerns, but are available from the corresponding author on reasonable request and in accordance with institutional policies.
