# Supplementary material for: Comparing emergency department use among individuals with varying levels of cognitive impairment
Source: BMC Geriatr. 2022 May 2;22:382. doi: 10.1186/s12877-022-03093-5 (PMC9059422; doi:10.1186/s12877-022-03093-5)
Supplement: Supplementary file 1 — Additional file 1: Appendix 1. Methods for Looking Through the Retrospectoscope. [file 12877_2022_3093_MOESM1_ESM.docx]

Appendix 1: Methods for Looking Through the Retrospectoscope

| Layers of Looking Through the Retrospectoscope | Evaluation of Appropriateness for this Study |
| --- | --- |
| Layer 1: Chart review appropriate for the research question | Patients were included in the study if they were evaluated in a UW Health memory clinic. This gave us access to the whole population being studied. All neuropsychological testing is consistently documented in charts at these visits, thus there was not a concern for missing data. |
| Layer 2: Transparency of investigator bias | No relevant conflicts of interest exist for this study. This study was approved by UW-Madison’s IRB. The data collection form was pilot-tested by RKG with feedback from MNS and went through several iterations before being put into practice. |
| Layer 3: Study and target population | All charts satisfying eligibility criteria were included in the study, diminishing selection bias. Inclusion/exclusion criteria were determined prior to chart reviews being conducted. Characteristics of the study population were similar to that of Dane County in general. The study was developed using the Strengthening the Reporting of Observational Studies in Epidemiology (STROBE) guidelines. |
| Layer 4: Variables to be collected | All variables abstracted from the chart were numerical or the verbatim diagnostic impression of the neuropsychologist performing the evaluation. This significantly reduced the likelihood of misinterpretation during abstraction. |
| Layer 5: Systematic data collection | Data was collected using a standardized Excel form and entered directly into a spreadsheet. |
| Layer 6: Missing and conflicting data | The diagnostic impression is the only variable used in the analysis to categorize patients into one of three groups. This variable was never missing. When it was unclear what the diagnostic impression meant or the diagnostic impression seemed to conflict with testing results, a combination of neuropsychologist review and ICD codes assigned at the visit was used to determine how to classify subjects. Clarification was needed in 75 cases. Neuropsychological testing scores are presented as descriptive characteristics of the population and were seldom missing. If it was still unclear how to classify a patient after review, they were excluded. |
| Layer 7: Abstractor bias | The chart abstractor (RKG) was not blinded to the study objectives and hypothesis, however as previously discussed, the abstracted variables were scores and verbatim diagnostic impressions, thus not subject to interpretation. The outcome variable was obtained through a third-party source, thus minimizing potential bias. |
| Layer 8: Abstractor training | The chart reviewer was a master’s student studying public health with no formal medical educational background. However, the chart reviewer was familiar with navigating electronic health records and was trained by the PI (MNS) on finding the appropriate notes with the needed information. |
| Layer 9: Abstractor monitoring | The PI (MNS) checked entries performed by the chart abstractor to ensure consistency and accuracy. Monitoring formally occurred twice over the two months of chart abstraction with no discrepancies found, however questionable entries were always flagged and brought to the PI for clarification. |
| Layer 10: Abstractor interrater reliability | Only one abstractor was used, thus Layers 10-10C are not applicable. |
